# Corrigendum to SUMOylation promotes extracellular vesicle–mediated transmission of lncRNA ELNAT1﻿ ﻿and lymph node metastasis in bladder cancer

**DOI:** 10.1172/JCI210810

**Published:** 2026-08-17

**Authors:** Changhao Chen, Hanhao Zheng, Yuming Luo, Yao Kong, Mingjie An, Yuting Li, Wang He, Bowen Gao, Yue Zhao, Hao Huang, Jian Huang, Tianxin Lin

Original citation: *J Clin Invest.* 2021;131(8):e146431. https://doi.org/10.1172/JCI146431

Citation for this corrigendum: *J Clin Invest.* 2026;136(16):e210810. https://doi.org/10.1172/JCI210810

The authors recently noticed that the representative 18S rRNA immunofluorescence image in Supplemental Figure 7B appeared to have nonspecific background staining for the nuclear signal of 18S rRNA. The authors newly repeated the experiment 3 additional times, and corrected the figure. The supplemental file has been updated online. The authors have stated that the new figure does not change the conclusions in the article.

The authors regret the errors.

## Supplementary Material

Supplemental data

## Version 1 08/17/2026

Electronic publication

