# Supplementary material for: Corrigendum to SUMOylation promotes extracellular vesicle–mediated transmission of lncRNA ELNAT1﻿ ﻿and lymph node metastasis in bladder cancer
Source: J Clin Invest. 2026 Aug 17;136(16):e210810. doi: 10.1172/JCI210810 (PMC13476139; doi:10.1172/JCI210810)
Supplement: Supplementary file 1 [file jci-136-210810-s127.pdf]

SUMOylation promotes extracellular vesicle-mediated transmission of lncRNA *ELNAT1* and lymph node metastasis in bladder cancer

Changhao Chen[1,2#*], Hanhao Zheng[1,2#], Yuming Luo[3#], Yao Kong[3], Mingjie An[1,2], Yuting Li[3], Wang He[1,2], Bowen Gao[4], Yue Zhao[5], Hao Huang[1,2], Jian Huang[1,2*], Tianxin Lin[1,2*]

[1]Department of Urology, Sun Yat-sen Memorial Hospital, Guangzhou, Guangdong, P. R. China

[2]Guangdong Provincial Key Laboratory of Malignant Tumor Epigenetics and Gene Regulation, Sun Yat-sen Memorial Hospital, State Key Laboratory of Oncology in South China, Guangzhou, Guangdong, P. R. China

[3]Department of General Surgery, Guangdong Provincial People's Hospital, Guangdong Academy of Medical Sciences, Guangzhou, Guangdong, P. R. China

[4]Department of Pancreatobiliary Surgery, Sun Yat-sen Memorial Hospital, Guangzhou, Guangdong, P. R. China

[5]Department of Tumor Intervention, Sun Yat-sen University First Affiliated Hospital, Guangzhou, Guangdong, P. R. China

*** Correspondence to:**

Tianxin Lin & Jian Huang & Changhao Chen. Department of Urology, Sun Yat-sen Memorial Hospital, 107 Yanjiangxi Road, Yuexiu District, Guangzhou, Guangdong Province, P. R. China, postal code: 510120; E-mail: lintx@mail.sysu.edu.cn & huangj8@mail.sysu.edu.cn & chenchh53@mail.sysu.edu.cn; Tel.: +86-13724008338, Work Tel.: +86-20-34070447; Fax: +86-20-81332336.

# These authors contributed equally to this study.

The authors declare no competing interests.

Supplemental Figures

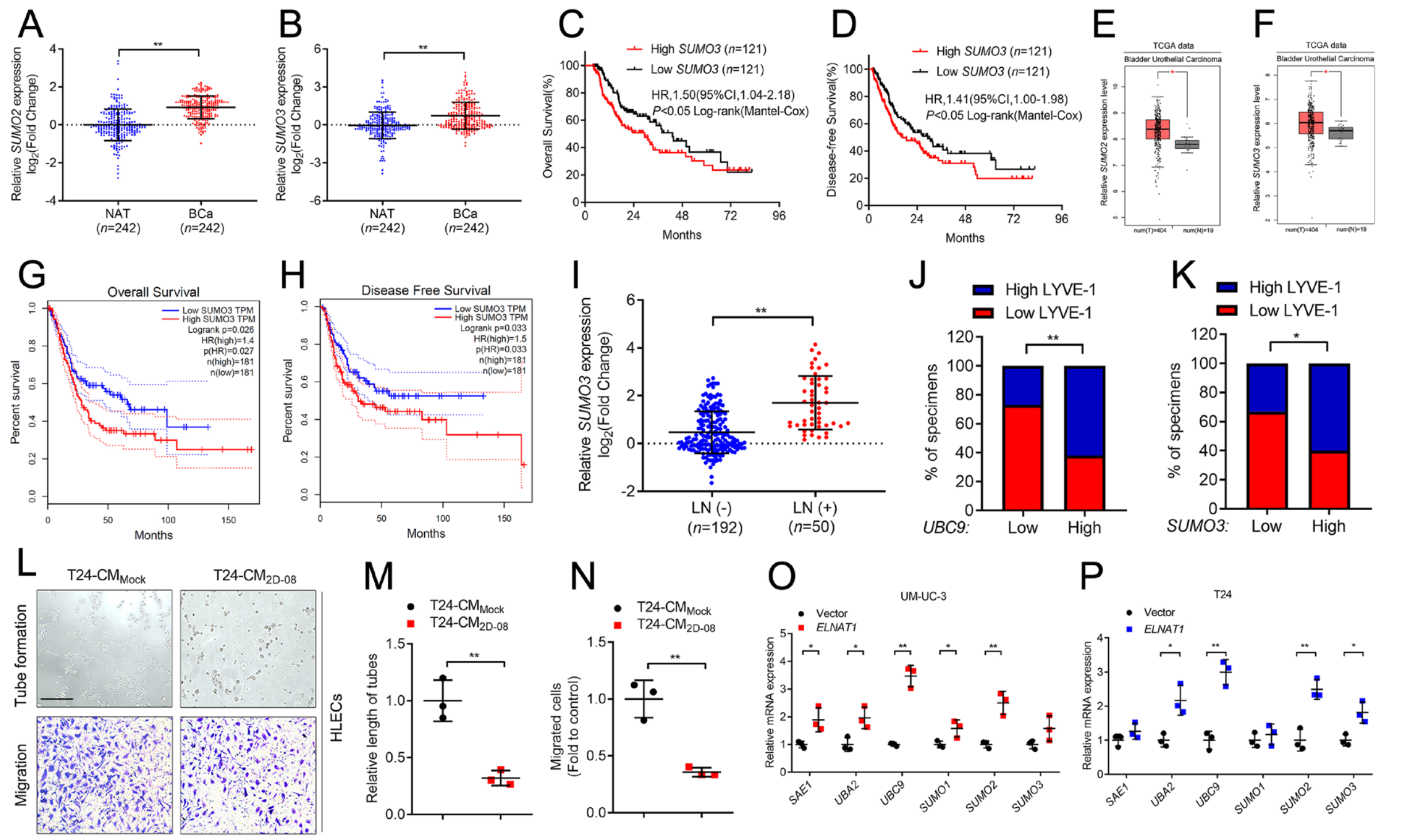

Supplemental Figure 1. SUMOylation is involved in the LN metastasis of BCa. (**A and B**) qRT-PCR analysis of SUMOylation core components *SUMO2* (A) and *SUMO3* (B) expression in BCa tissues and paired NATs in a 242-case cohort of BCa patients. The Nonparametric Mann-Whitney *U* test was used to assess the statistical significance. (**C and D**) Kaplan-Meier curves for the OS and DFS of BCa patients with low vs. high *SUMO3* expression (cutoff value is the median). (**E and F**) The expression of *SUMO2* (E) and *SUMO3* (F) in BCa patients compared with controls from TGCA database. (**G and H**) Kaplan-Meier survival analysis of BCa patients according to *SUMO3* expression from TCGA database (cutoff value is the best cutoff). (**I**) qRT-PCR analysis of *SUMO3* expression in LN-positive and LN-negative BCa tissues (n=242). The Nonparametric Mann-Whitney *U* test was used to assess the statistical significance. (**J and K**) The percentages of IHC staining for the lymphatic vessel density in BCa tissues according to *UBC9* (J) and *SUMO3* (K) expression. $\chi 2$ test was used to assess the statistical significance. (**L-N**) Representative

images and quantification of tube formation and Transwell migration for HLECs incubation with culture media from T24 cells treated with PBS or SUMOylation inhibitor, 2D-08. Scale bars: 100 μm. Two-tailed Student's *t* test was used to assess the statistical significance. (**O and P**) qRT-PCR analysis of SUMOylation components in *ELNAT1* overexpressing BCa cells. Two-tailed Student's *t* test was used to assess the statistical significance. Error bars showed the SD of three independent experiments. *$P < 0.05$; **$P < 0.01$.

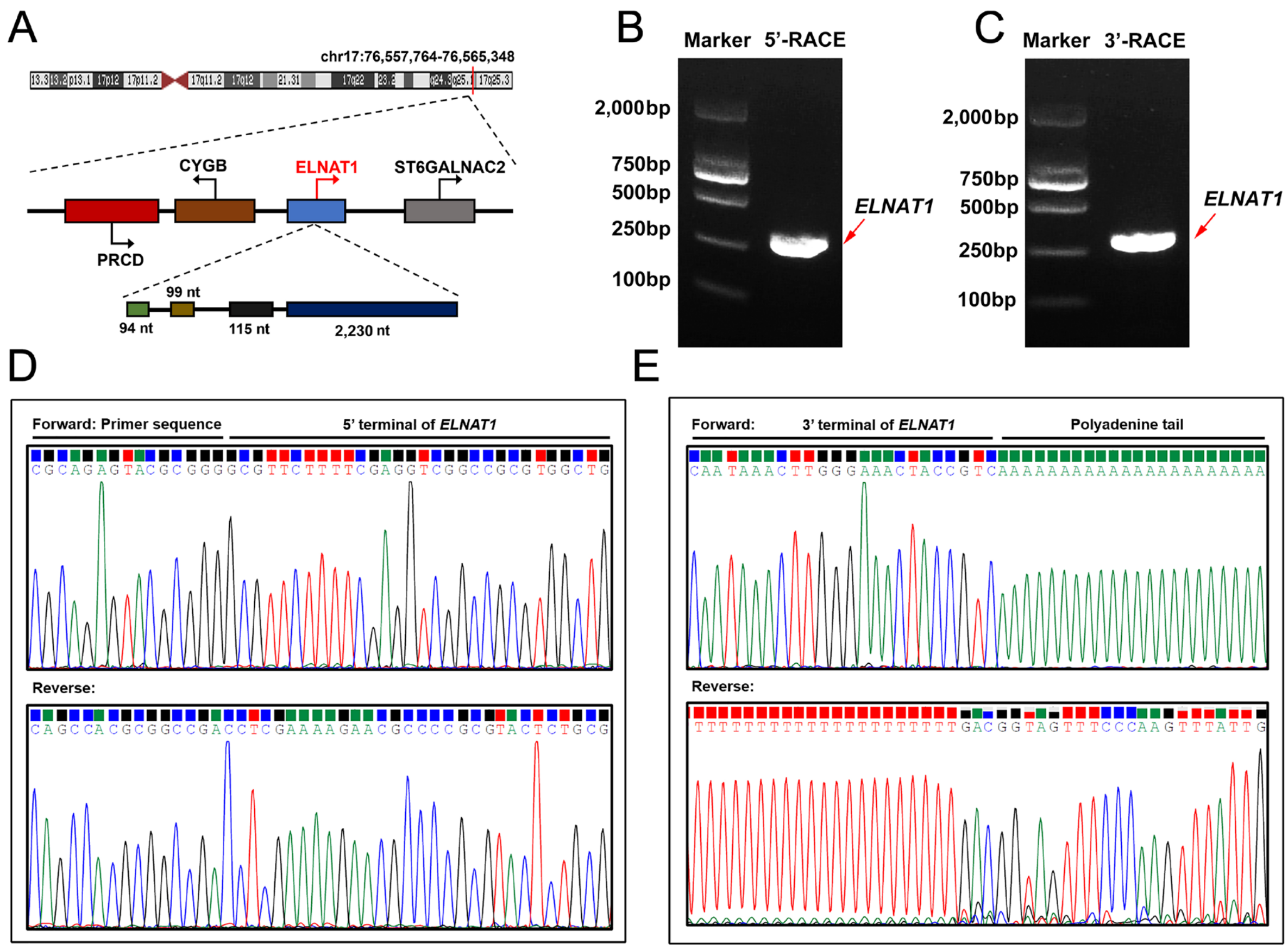

Supplemental Figure 2. Identification of the full-length of *ELNAT1*. (**A**) Schematic illustration for the genomic locus of *ELNAT1* on chromosome and the neighboring protein coding genes. (**B-E**) Representative images of agarose gel electrophoresis and bidirectional sequencing for the cap sequences, 5'-terminal sequences of *ELNAT1* obtained from 5'-RACE PCR (B and D) and the tail sequences, 3'-terminal sequences of *ELNAT1* obtained from 3'-RACE PCR (C and E).

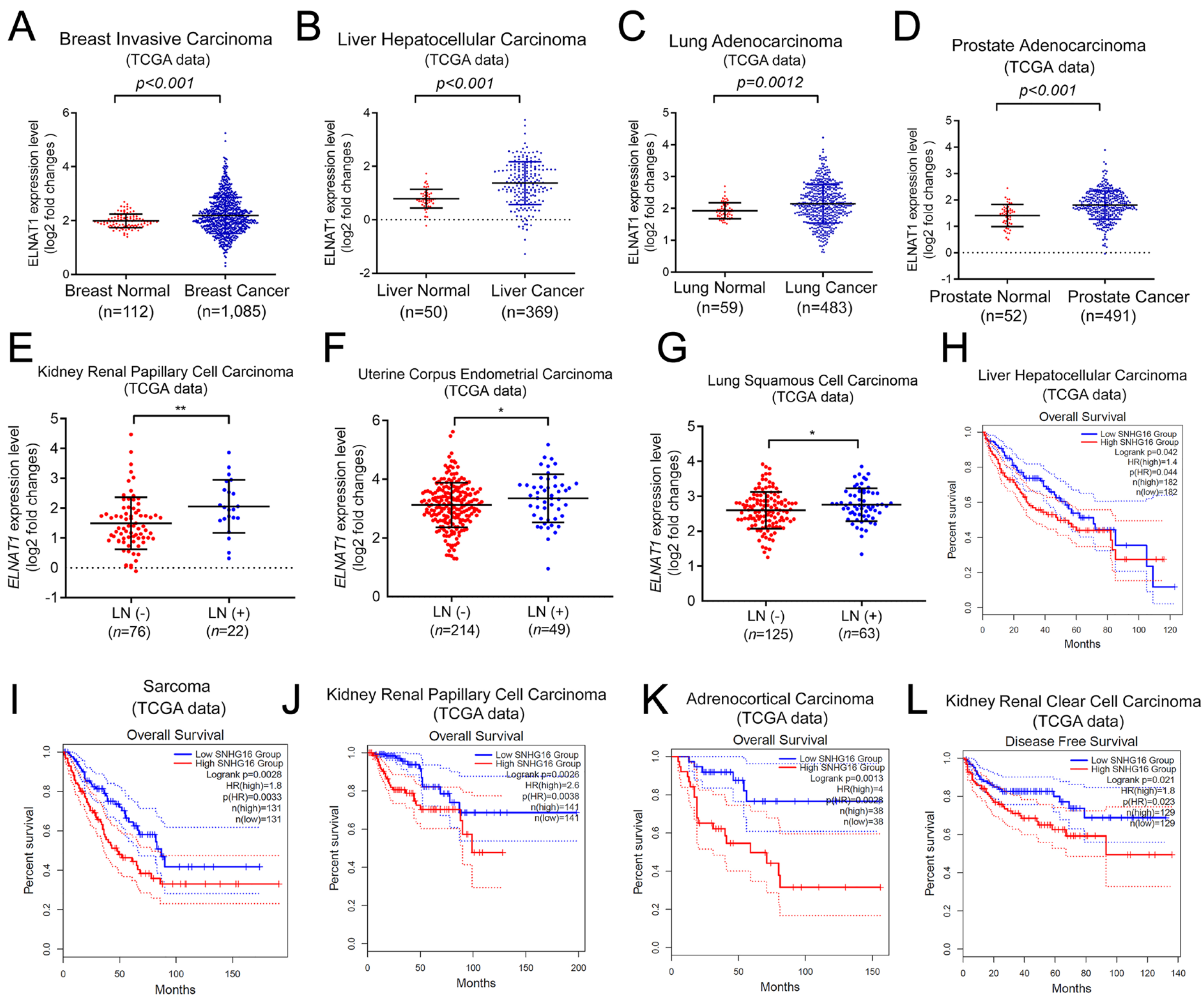

Supplemental Figure 3. *ELNAT1* is overexpressed in various human cancers and correlates with poor prognosis. (**A-D**) Analysis of TCGA database showed the *ELNAT1* expression in different types of human cancers compared with corresponding control. The Nonparametric Mann-Whitney *U* test was used to assess the statistical significance. **(E-G)** Analysis of TCGA database revealed the higher *ELNAT1* expression in LN-positive tumor tissues as compared with LN-negative tissues in multiple cancers. The Nonparametric Mann-Whitney *U* test was used to assess the statistical significance. **(H-L)** Kaplan-Meier survival analysis of patients according to *ELNAT1* expression in various types of cancers from TCGA database (cutoff value is the best cutoff). $*P < 0.05$, $**P < 0.01$.

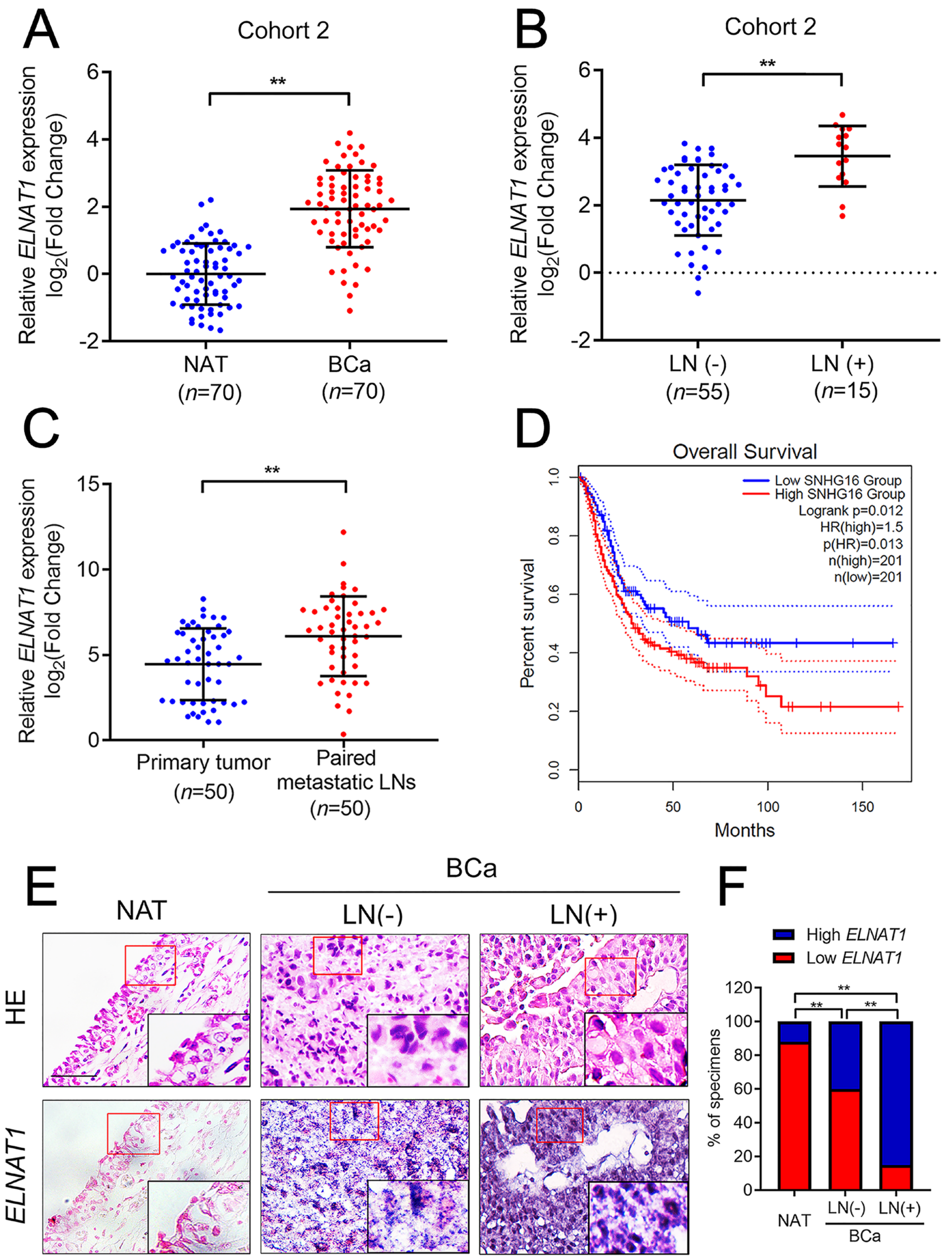

Supplemental Figure 4. EV-mediated *ELNAT1* overexpression correlates with LN metastasis of BCa. (A and B) qRT-PCR analysis of *ELNAT1* expression in BCa tissues and NATs (A), LN-positive and LN-negative BCa tissues (B) in another 70-case cohort of BCa

patients. The Nonparametric Mann-Whitney *U* test was used to assess the statistical significance. (**C**) qRT-PCR analysis of *ELNAT1* expression in primary tumors and paired metastatic LNs in BCa patients (n=50). The Nonparametric Mann-Whitney *U* test was used to assess the statistical significance. (**D**) Kaplan-Meier curves for BCa patients according to *ELNAT1* expression from TCGA database (cutoff value is the best cutoff). (**E and F**) Representative ISH images and percentages for *ELNAT1* expression in LN-positive or LN-negative BCa tissues and NATs. Scale bars: 50 μm. $\chi 2$ test was used to assess the statistical significance. *$P < 0.05$, **$P < 0.01$.

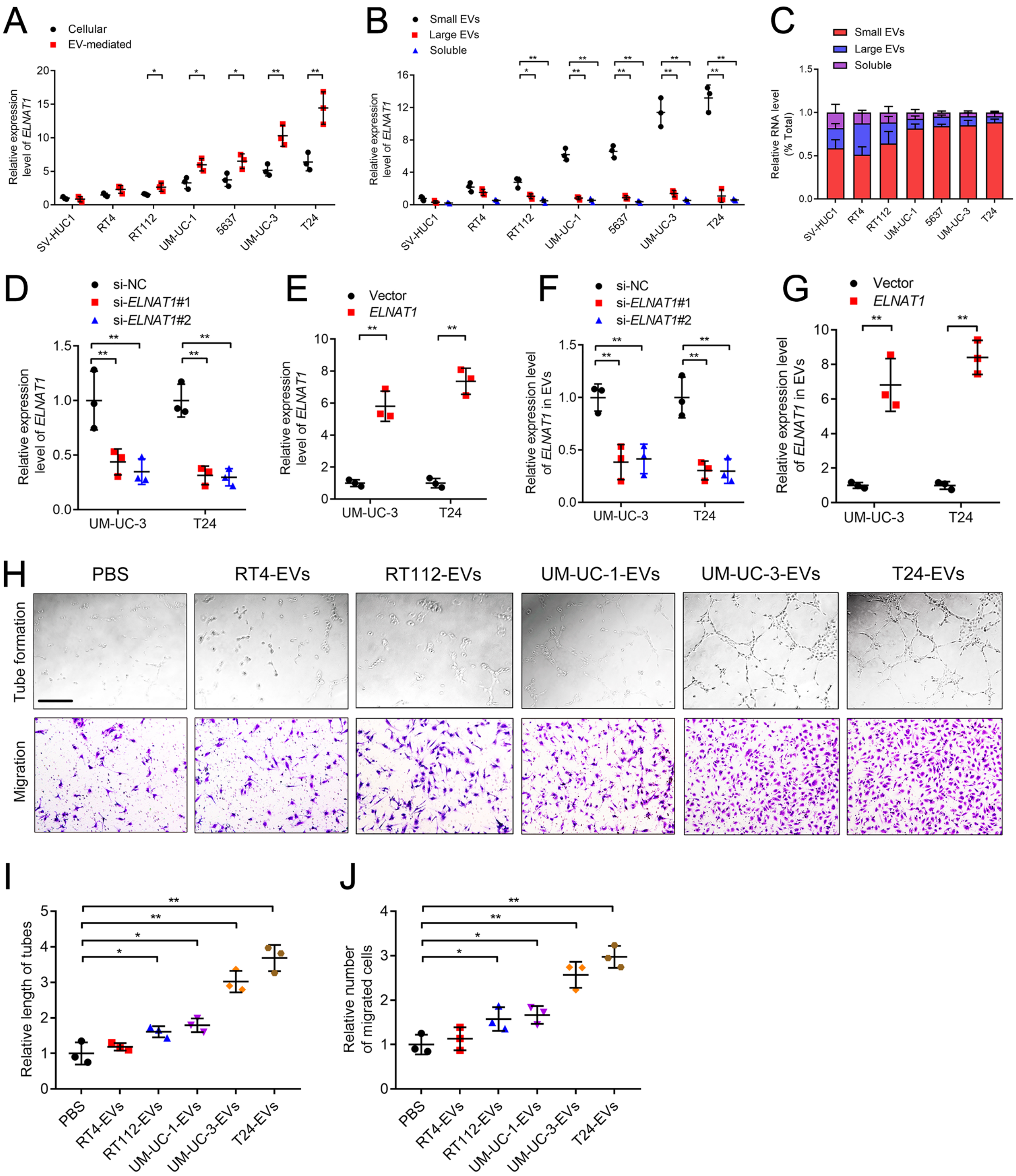

Supplemental Figure 5. *ELNAT1* is overexpressed in BCa cells-secreted EVs. (A) qRT-PCR analysis of *ELNAT1* expression in indicated bladder cell lines and their corresponding EVs. 1-way ANOVA followed by Dunnett's tests were used to assess the statistical significance. (**B and C**) The *ELNAT1* expression and percentages in different extracellular forms of indicated bladder cell lines are evaluated by qRT-PCR analysis. 1-way

ANOVA followed by Dunnett's tests were used to assess the statistical significance. (**D-G**) qRT-PCR analysis of the *ELNAT1* expression in BCa cell lines and corresponding EVs after silencing or overexpressing *ELNAT1*. 1-way ANOVA followed by Dunnett's tests or Two-tailed Student's t-test were used to assess the statistical significance. **(H-J)** Representative images and quantification of tube formation and Transwell migration for HLECs treated with BCa cells-secreted EVs. Scale bars: 100 μm. 1-way ANOVA followed by Dunnett's tests were used to assess the statistical significance. Error bars showed the SD of three independent experiments. *$P < 0.05$, **$P < 0.01$.

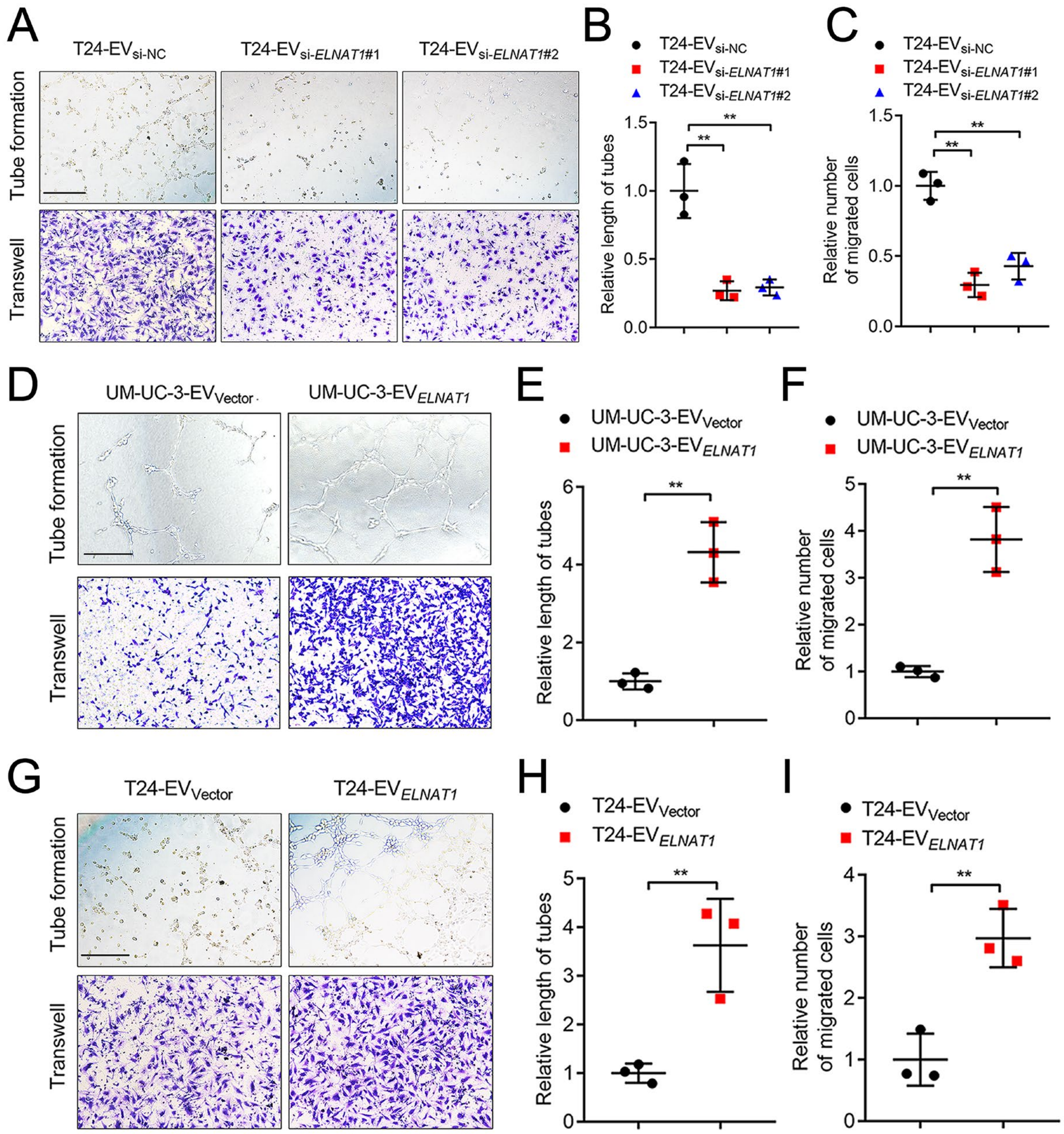

Supplemental Figure 6. EV-mediated *ELNAT1* promotes lymphangiogenesis of BCa in vitro. (**A-C**) Representative images and quantification of tube formation and Transwell migration for HLECs treated with T24-EV$_{\text{si-NC}}$, T24-EV$_{\text{si-}\textit{ELNAT1}\#1}$ or T24-EV$_{\text{si-}\textit{ELNAT1}\#2}$. Scale bars: 100 µm. 1-way ANOVA followed by Dunnett's tests were used to assess the statistical significance. (**D-F**) Representative images and quantification of tube formation and Transwell migration for HLECs treated with UM-UC-3-EV$_{\text{Vector}}$ or UM-UC-3-EV$_{\textit{ELNAT1}}$. Scale bars: 100 µm. Two-tailed Student's *t*-test was used to assess the statistical significance.

(**G-I**) Representative images and quantification of tube formation and Transwell migration for HLECs treated with T24-EV_{Vector} or T24-EV_{ELNAT1}. Scale bars: 100 μm. Two-tailed Student's *t*-test was used to assess the statistical significance. Error bars showed the SD of three independent experiments. **P* < 0.05, ***P* < 0.01.

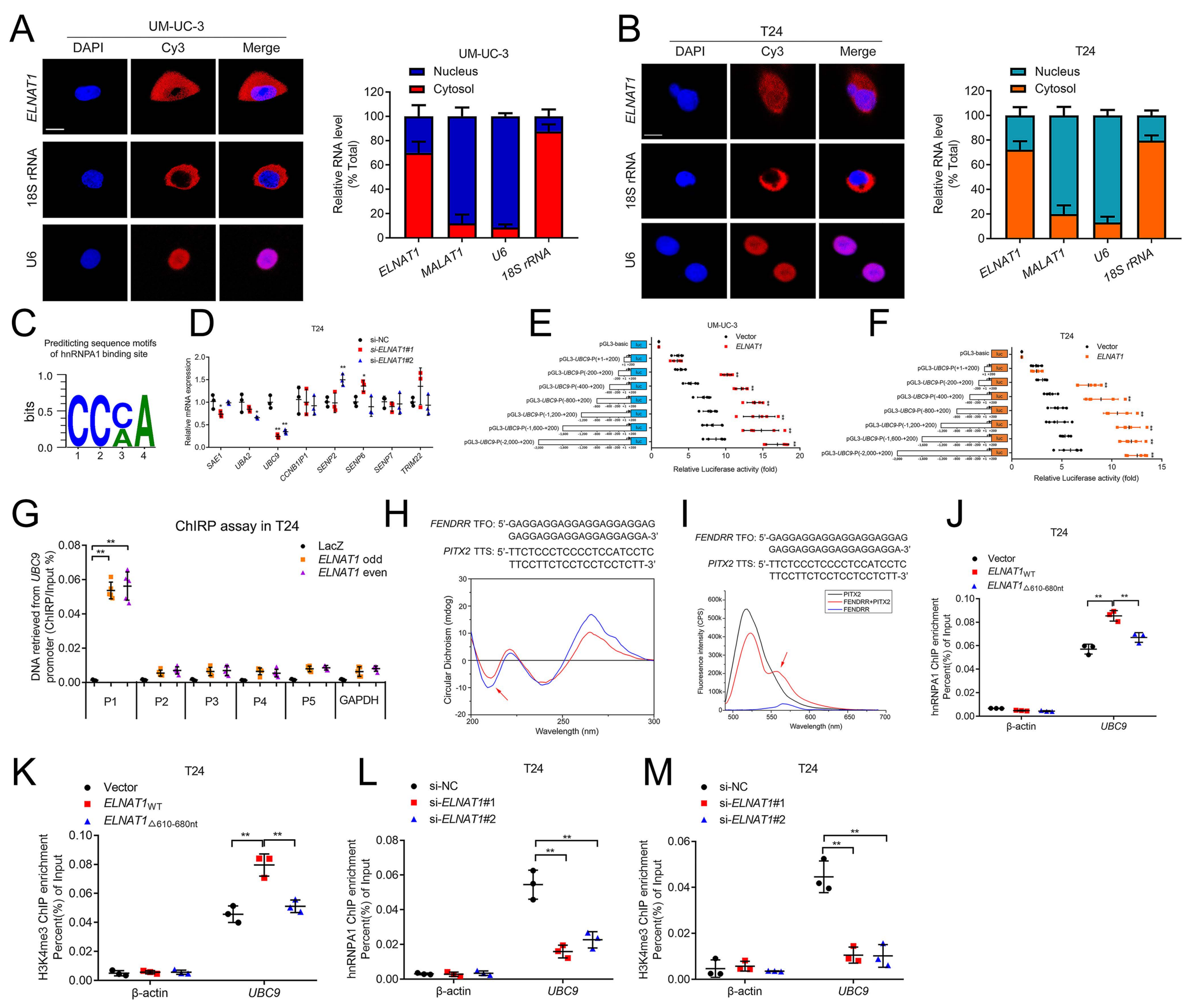

Supplemental Figure 7. *ELNAT1* enhances H3K4me3 modification by recruiting hnRNPA1 in *UBC9* promoter. (A and B) FISH assays and subcellular fractionation assays of *ELNAT1* in UM-UC-3 or T24 cells. Scale bar: 5 μm. (**C**) Prediction for the sequence motifs of hnRNPA1 binding sites in *ELNAT1*. (**D**) qRT-PCR analysis of the SUMOylation-related genes after *ELNAT1* knockdown in T24 cells. 1-way ANOVA followed

by Dunnett's tests were used to assess the statistical significance. (**E and F**) Luciferase assays for the truncated *UBC9* promoter after *ELNAT1* overexpressing in UM-UC-3 and T24 cells. Two-tailed Student's *t*-test was used to assess the statistical significance. **(G)** ChIRP analysis of *ELNAT1*-associated chromatin in T24 cells. 1-way ANOVA followed by Dunnett's tests were used to assess the statistical significance. (**H**) CD spectrum of TFO in *FENDRR* with TTS in *PITX2* promoter was examined as positive control. (**I**) FRET analysis of TFO in *FENDRR* with TTS in *PITX2* promoter was examined as positive control. (**J-M**) ChIP-qPCR analysis of the hnRNPA1 occupancy and H3K4me3 status in *UBC9* promoter in indicated T24 cells. 1-way ANOVA followed by Dunnett's tests were used to assess the statistical significance. Error bars showed the SD of three independent experiments. $*P < 0.05$, $**P < 0.01$.

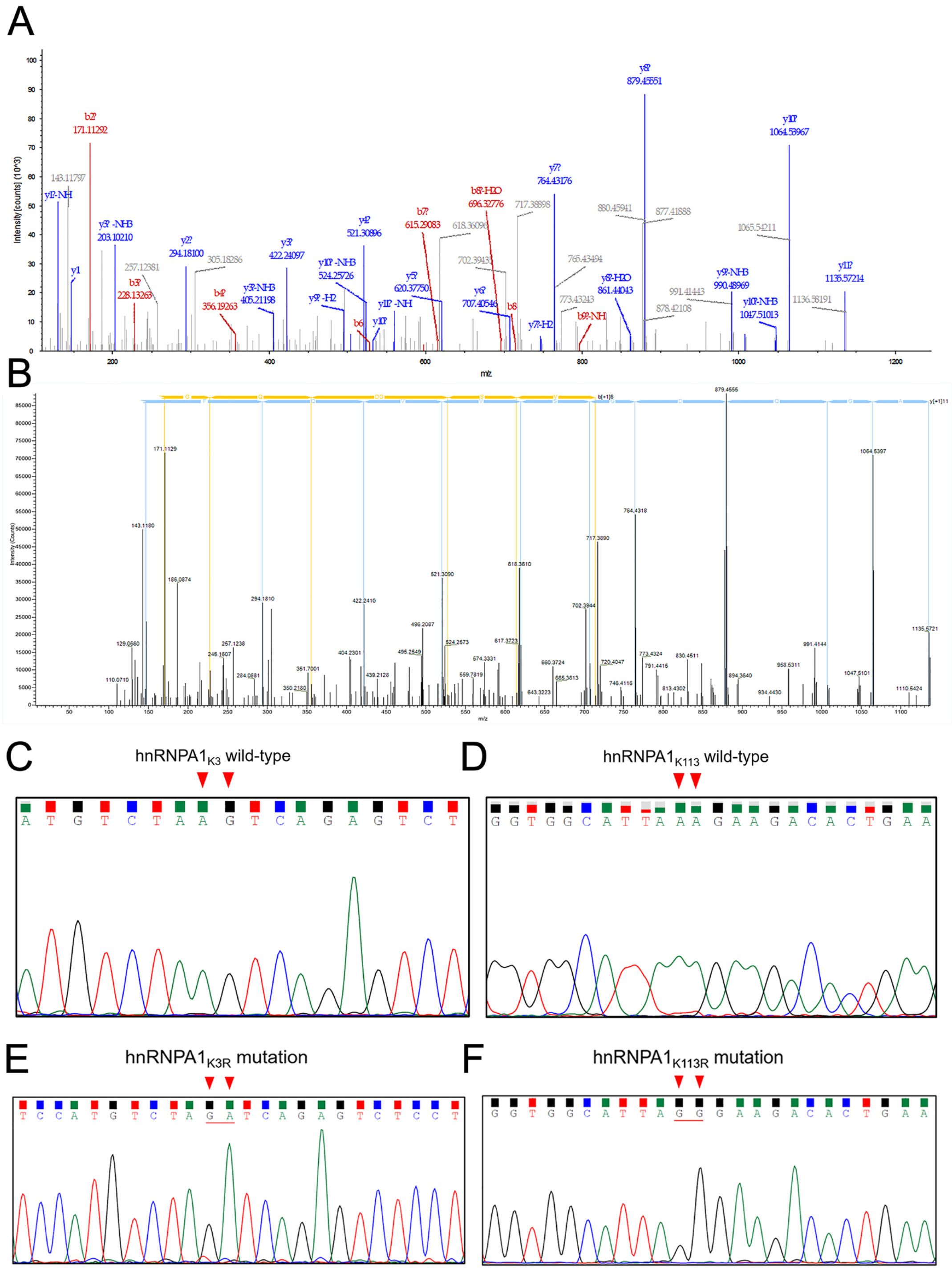

Supplemental Figure 8. HnRNPA1 is SUMOylated at its lysine-113 residue. (**A and B**) Mass spectrometry analysis of the proteins obtained from co-IP assay using anti-hnRNPA1 in UM-UC-3 cells. (**C and D**) Sequencing of the hnRNPA1$_{K3}$ and hnRNPA1$_{K113}$ wild type. (**E and F**) Sequencing of the hnRNPA1$_{K3R}$ and hnRNPA1$_{K113R}$ mutations.

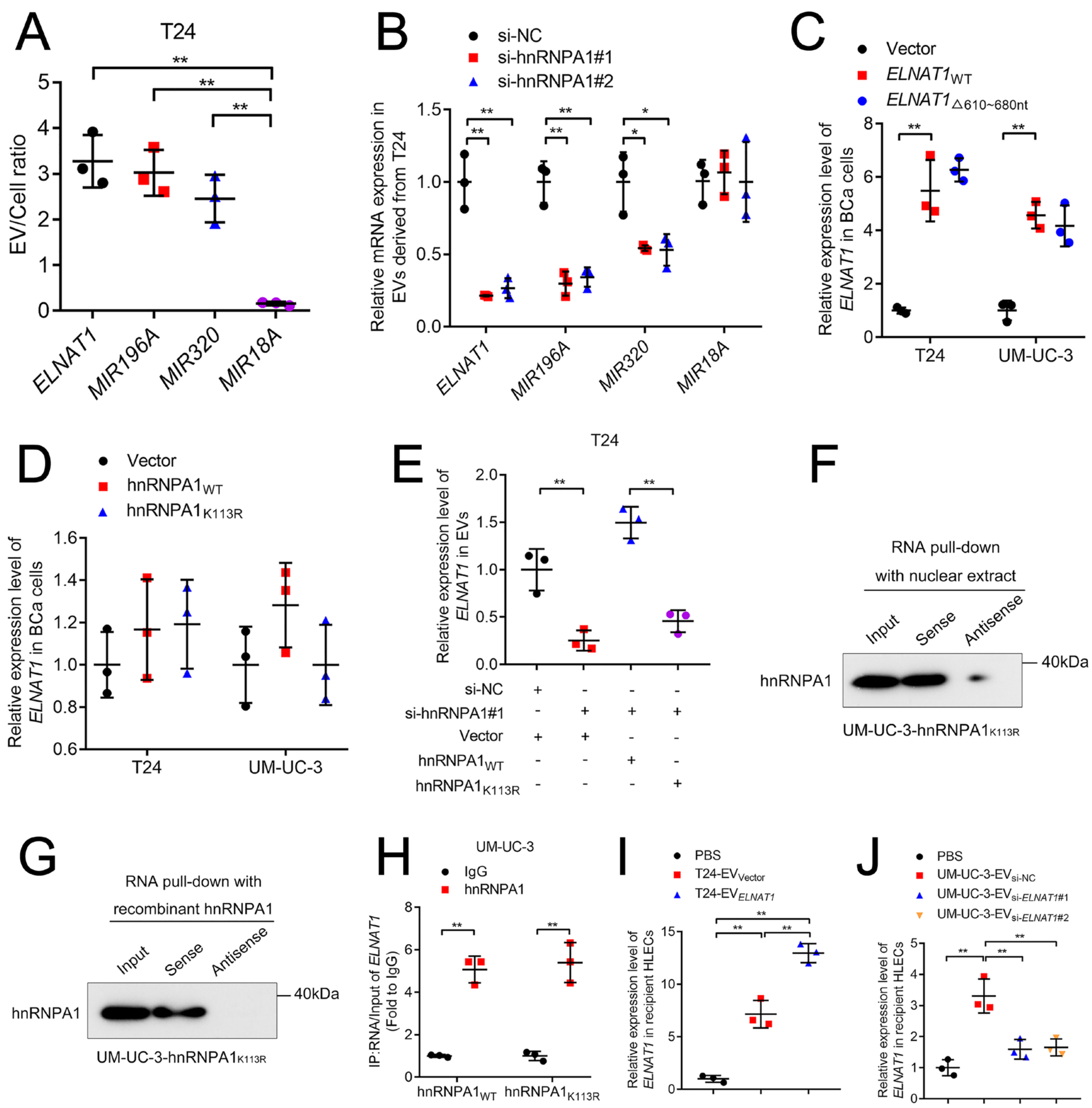

Supplemental Figure 9. *ELNAT1* is packaged into EVs by SUMOylation of hnRNPA1.

(**A**) The EV/cell ratio of RNAs in T24 cells. 1-way ANOVA followed by Dunnett's tests were used to assess the statistical significance. (**B**) qRT-PCR analyzed the RNAs expression in EVs secreted by T24 cells after hnRNPA1 knockdown. 1-way ANOVA followed by Dunnett's tests were used to assess the statistical significance. (**C**) The assessment of *ELNAT1* expression in BCa cells after the deletion of 610-680-nt of *ELNAT1*. 1-way ANOVA followed by Dunnett's tests were used to assess the statistical significance. (**D**) The analysis of *ELNAT1* expression in BCa cells after hnRNPA1$_{K113R}$ mutation. 1-way ANOVA

followed by Dunnett's tests were used to assess the statistical significance. (**E**) The *ELNAT1* expression in EVs secreted by hnRNPA1-knockdown T24 cells after $hnRNPA1_{WT}$ or $hnRNPA1_{k113R}$ overexpression was assessed by qRT-PCR analysis. 1-way ANOVA followed by Dunnett's tests were used to assess the statistical significance. (**F and G**) RNA pull-down and western blotting analyses with nuclear extract or purified recombinant hnRNPA1 evaluated the interaction between *ELNAT1* and hnRNPA1 after $hnRNPA1_{K113R}$ mutation in UM-UC-3 cells. (**H**) RIP assays using anti-hnRNPA1 assessed the enrichment of *ELNAT1* by hnRNPA1 after $hnRNPA1_{K113R}$ mutation in UM-UC-3 cells. IgG: negative control; U1: nonspecific control. Two-tailed Student's *t*-test was used to assess the statistical significance. (**I and J**) qRT-PCR analysis of *ELNAT1* expression in HLECs treated with indicated BCa cells-secreted EVs. 1-way ANOVA followed by Dunnett's tests were used to assess the statistical significance. Error bars show the SD of three independent experiments. $*P < 0.05$, $**P < 0.01$.

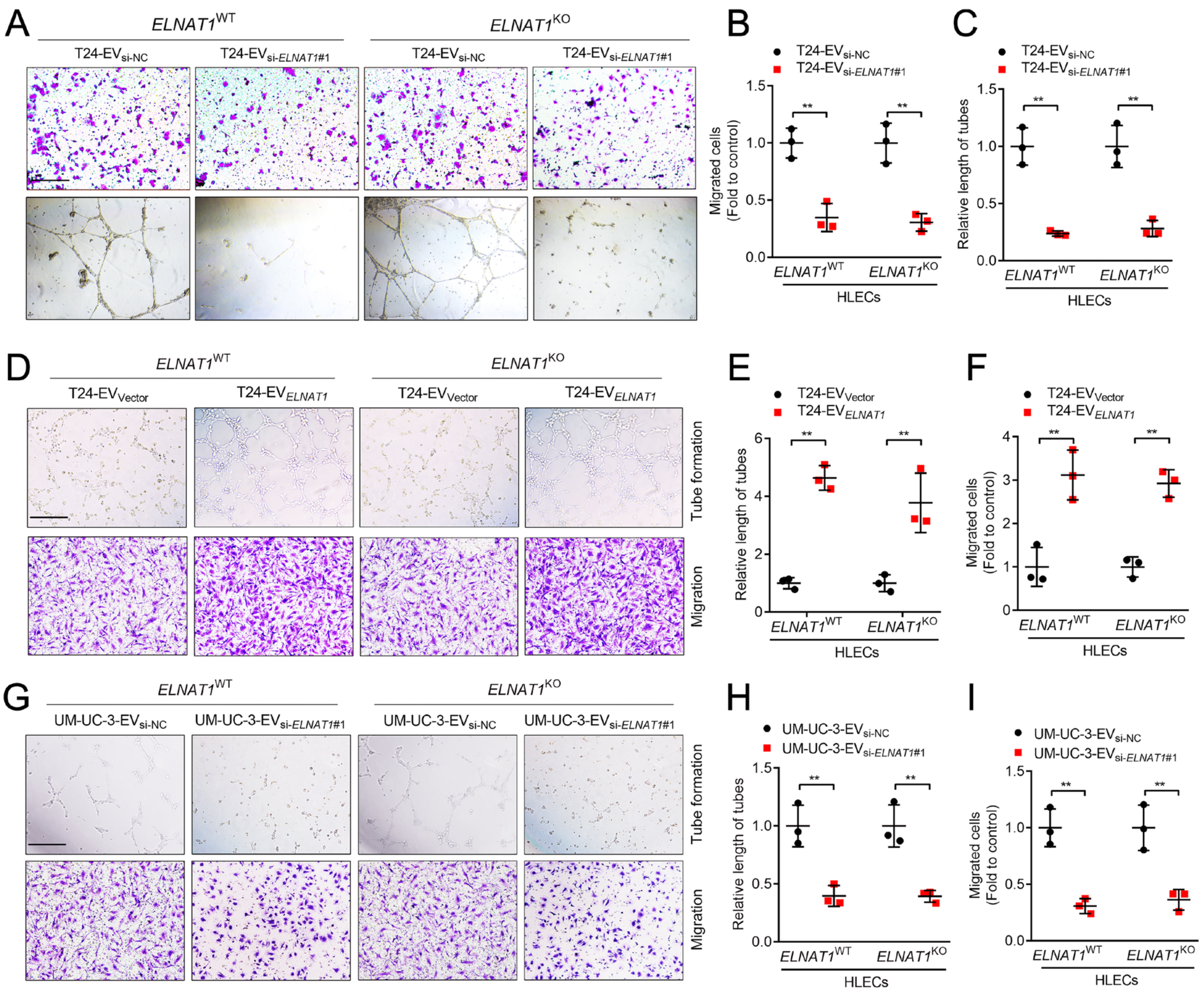

Supplemental Figure 10. BCa cell-secreted EVs induces lymphangiogenesis through transmitting *ELNAT1*. (**A-C**) Representative images and quantification of Transwell migration and tube formation for *ELNAT1*^{WT} or *ELNAT1*^{KO} HLECs treated with T24-EV$_{si\text{-}NC}$ or T24-EV$_{si\text{-}ELNAT1\#1}$. Scale bars: 100 µm. Two-tailed Student's *t*-test was used to assess the statistical significance. (**D-F**) Representative images and quantification of tube formation and Transwell migration for *ELNAT1*^{WT} or *ELNAT1*^{KO} HLECs treated with T24-EV$_{Vector}$ or T24-EV$_{ELNAT1}$. Scale bars: 100 µm. Two-tailed Student's *t*-test was used to assess the statistical significance. (**G-I**) Representative images and quantification of tube formation and Transwell migration for *ELNAT1*^{WT} or *ELNAT1*^{KO} HLECs treated with UM-UC-3-EV$_{si\text{-}NC}$, UM-UC-3-EV$_{si\text{-}ELNAT1\#1}$. Scale bars: 100 µm. Two-tailed Student's *t*-test was used to assess

the statistical significance. Error bars show the SD of three independent experiments. *$P <$ 0.05, **$P < 0.01$.

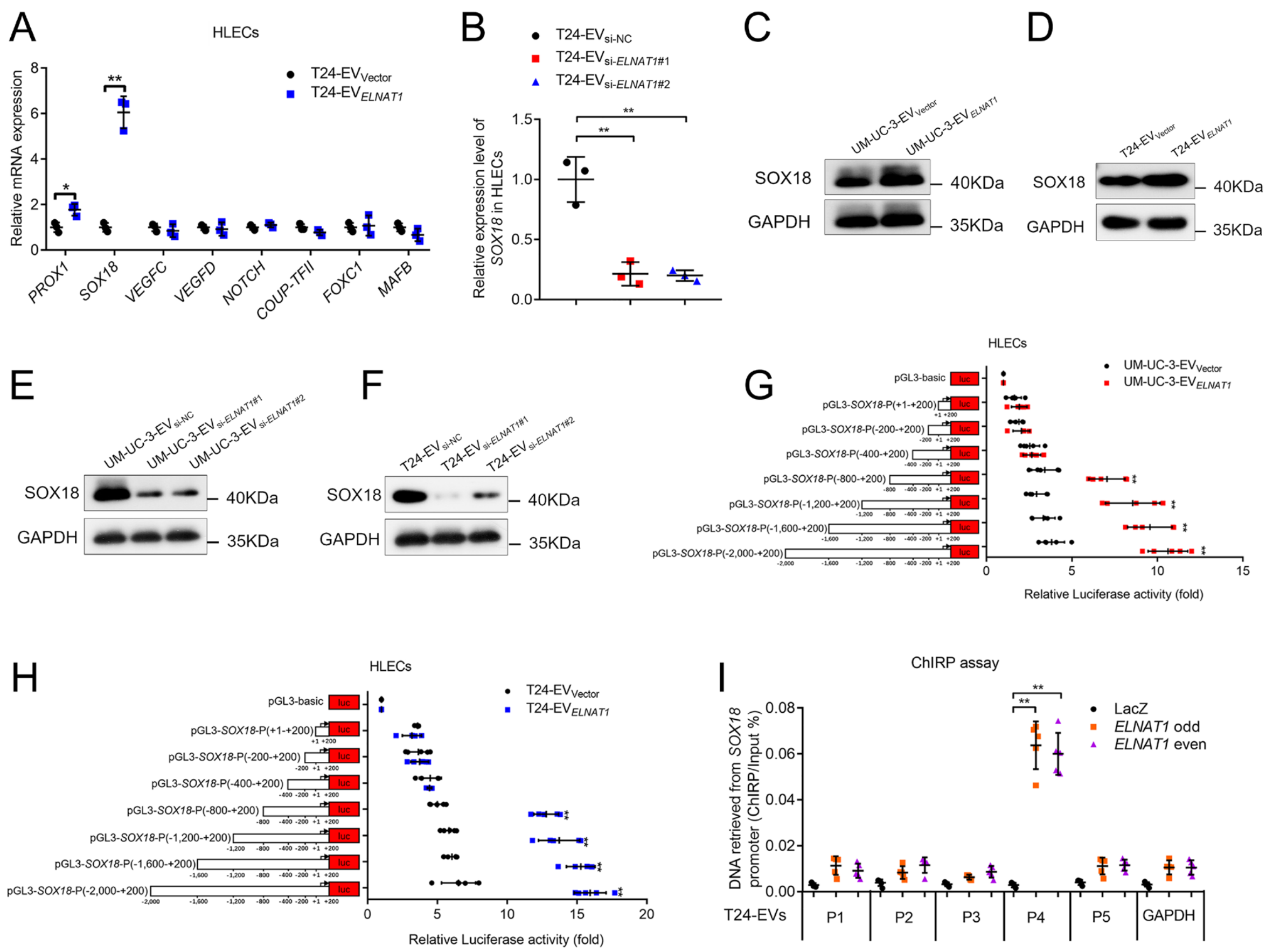

Supplemental Figure 11. EV-mediated *ELNAT1* upregulates SOX18 expression in HLECs. (**A**) qRT-PCR analysis of lymphangiogenesis-related genes expression in T24 $_{\text{Vector}}$ or T24-EV$_{ELNAT1}$-treated HLECs. Two-tailed Student's *t*-test was used to assess the statistical significance. (**B**) qRT-PCR analysis of *SOX18* expression in HLECs treated with T24-EV$_{\text{si-NC}}$, T24-EV$_{\text{si-}ELNAT1\#1}$ or T24-EV$_{\text{si-}ELNAT1\#2}$. 1-way ANOVA followed by Dunnett's tests were used to assess the statistical significance. (**C-F**) Western blotting analysis of SOX18 expression in HLECs treated with indicated BCa cells-secreted EVs. (**G and H**) Luciferase assays for the serial deletion of *SOX18* promoter in HLECs treated with UM-UC-3-EV$_{\text{Vector}}$, UM-UC-3-EV$_{ELNAT1}$ or T24-EV$_{\text{Vector}}$, T24-EV$_{ELNAT1}$. Two-tailed Student's *t*-test was used to assess the statistical significance. (**I**) ChIRP analysis of EV-mediated *ELNAT1*-associated

chromatin in T24-EVs-treated HLECs. 1-way ANOVA followed by Dunnett's tests were used to assess the statistical significance. Error bars show the SD of three independent experiments. *$P < 0.05$, **$P < 0.01$.

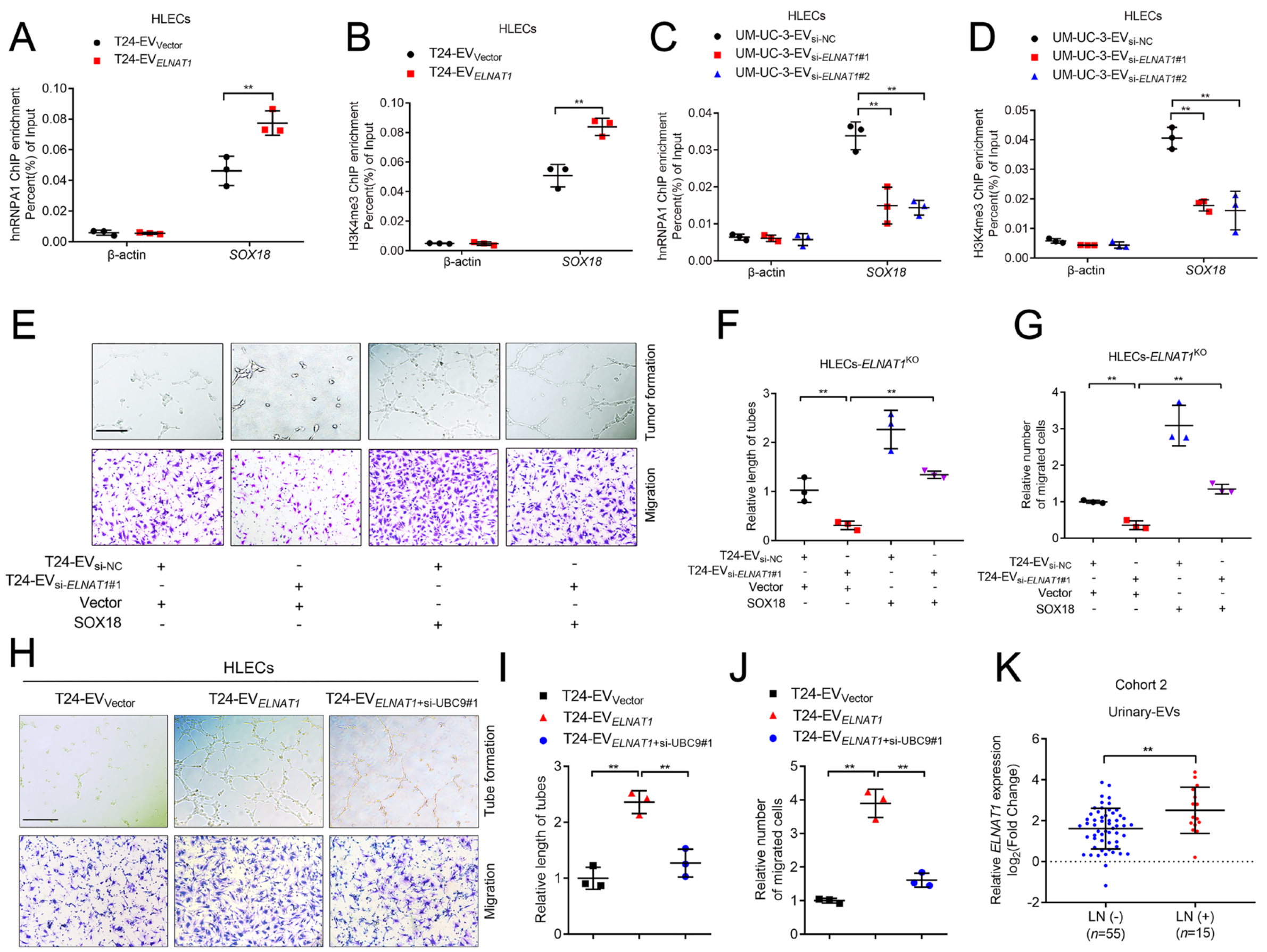

Supplemental Figure 12. Blocking SUMOylation suppresses EV-mediated *ELNAT1*-induced lymphangiogenesis. (**A-D**) ChIP-qPCR analysis of the hnRNPA1 occupancy and H3K4me3 status in the promoter of *SOX18* in HLECs treated with indicated BCa cells-secreted EVs. Two-tailed Student's *t*-test or 1-way ANOVA followed by Dunnett's tests were used to assess the statistical significance. (**E-G**) Representative images and quantification of tube formation and Transwell migration for T24-EV$_{\text{si-NC}}$ or T24-EV$_{\text{si-}ELNAT1\#1}$-treated *ELNAT1*^{KO} HLECs transfected with Vector or SOX18 plasmid. Scale bars: 100 μm. 1-way ANOVA followed by Dunnett's tests were used to assess the statistical significance. (**H-J**) Representative images and quantification of tube formation and

Transwell migration for HLECs treated with EVs secreted by control or *ELNAT1*-overexpressing T24 cells transfected with si-NC or si-UBC9#1. Scale bars: 100 μm. 1-way ANOVA followed by Dunnett's tests were used to assess the statistical significance. (**K**) qRT-PCR analysis of *ELNAT1* expression in urinary EVs obtained from another 70-case cohort of BCa patients with or without LN metastasis. The Nonparametric Mann-Whitney *U* test was used to assess the statistical significance. Error bars show the SD of three independent experiments. $*P < 0.05$, $**P < 0.01$.

Supplemental Tables

Supplemental Table 1. Patients with MIBC and healthy participants characteristics for next-generation sequencing (NGS) on urinary EVs.

Characteristics	Gender	Age	TNM stage	Pathological grade
BCa patient 1	Female	64	T3aN0M0	IV
BCa patient 2	Male	64	T2bN1M0	IV
BCa patient 3	Male	67	T3bN0M0	IV
BCa patient 4	Female	56	T2aN1M0	III
BCa patient 5	Male	48	T2aN0M0	III
Healthy participant 1	Female	60		
Healthy participant 2	Male	63		
Healthy participant 3	Male	68		
Healthy participant 4	Female	56		
Healthy participant 5	Male	51		

Abbreviations: BCa = bladder cancer; TNM stage = tumor node metastasis stage.

Supplemental Table 2. The lncRNAs co-upregulated in urinary-EVs from BCa patients and LN-positive BCa tissues.

Gene Symbol	Chromosome	Location
RP11-563N4.1	2	chr2:32,072,515-32,235,206
ELNAT1	17	chr17:76,557,764-76,565,348
RP11-357H14.17	17	chr17:48,502,127-48,689,498
MIR4435-2HG	2	chr2:111,006,015-111,523,376
ENSG00000275234	19	chr19:6,469,465-6,470,152
LINC00665	19	chr19:36,313,067-36,331,770
UCA1	19	chr19:15,828,206-15,836,328
ENSG00000279365	21	chr21:36,485,867-36,487,760
RP11-1079K10.4	17	chr17:49,292,915-49,476,604
LINC00858	10	chr10:84,279,980-84,294,659
ENSG00000229021	1	chr1:151,994,531-152,042,774
PVT1	8	Chr8:127,794,526-128,187,101

Supplemental Table 3. Effect of EV-mediated *ELNAT1* on popliteal LN metastasis in vivo.

Xenograft	No. metastasis LNs	No. Non-metastasis LNs	Metastasis ratio	*P*-value[A]
UM-UC-3-EV_{Vector}	3	9	25%	0.001^{**}
UM-UC-3-EV_{ELNAT1}	11	1	91.67%	

[A] Chi-square test. * p <0.05, ** p <0.01.

Supplemental Table 4. The change of SUMOylation-related genes after *ELNAT1* overexpressing in indicated BCa cells.

Gene Symbol	Location	Fold Change	*p*-value
SAE1	chr19:47,113,274-47,210,636	2.197512	0.000178
UBA2	chr19:34,428,352-34,471,251	2.117072	0.000405
UBC9	chr16:1,308,880-1,327,018	2.286423	0.000568
CCNB1IP1	chr14:20,311,368-20,333,312	1.673057	0.004854
SENP6	chr6:75,601,509-75,718,281	0.439591	0.001250
SENP2	chr3:185,582,496-185,633,551	0.420276	0.006155
SENP7	chr3:101,324,205-101,513,241	0.395299	0.016816
TRIM22	chr11:5,689,697-5,737,089	0.410256	0.048322

Supplemental Table 5. The possible TFO predicted by LongTarget for *ELNAT1* and UBC9 promoter.

Oligo ID	TFO (5'-3')	Oligo ID	TTS (5'-3')	Score
**TFO1**	GGUUGCCAAGGUGAAGC	TTS1	CCCGCCGTCGGCGAAAT	66
**TFO2**	CUCCCGGACUUAAGCGA	TTS2	GAGGGAATGAGTGAGGG	49
**TFO3**	CUCUGUCACCCAGGCUG	TTS3	GTGAGAGTGGGAATGAG	55
**TFO4**	CUGCCUGCCUCAGCUUC	TTS4	GAGGGAGGGAATGAATG	37
**TFO5**	CUUGGCUCACUGCAACC	TTS5	GAAGGGAATGAGTGAGG	44

Abbreviations: TFO, Triplex-forming oligos; TTS, triplex target sites.

Supplemental Table 6. The popliteal LN metastasis rate in differently treated group of nude mice.

Xenograft	No. metastasis LNs	No. Non-metastasis LNs	Metastasis ratio	*P*-value[A]
UM-UC-3-EV$_{Vector}$	2	10	16.67%	0.004^{**}
UM-UC-3-EV$_{\mathit{ELNAT1}}$	9	3	75%	0.041^{*}
UM-UC-3-EV$_{\mathit{ELNAT1}\text{+si-UBC9}}$	4	8	33.33%	

[A] Chi-square test. * p <0.05, ** p <0.01.

Supplemental Table 7. Correlation between EV-mediated *ELNAT1* expression and clinicopathologic characteristics of BCa patients

Characteristics	No. of cases	EV-mediated *ELNAT1* expression		
		**Low**	**High**	***P*-value[i]**
**Total cases**	242	121	121	
**Gender**				0.882
Male	181	91	90	
Female	61	30	31	
**Age**				0.298
< 65	102	55	47	
≥ 65	140	66	74	
**T stage**				0.075
T1	79	46	33	
T2-4	163	75	88	
**T grade**				0.310
Low	65	29	36	
High	177	92	85	
**Lymphatic metastasis**				**0.001****
Negative	192	111	81	
Positive	50	10	40	

Abbreviations: No. of cases = number of cases; T stage = tumor stage; T grade = tumor grade.

[i] Chi-square test, * $P<0.05$, ** $P<0.01$.

Supplemental Table 8. Univariate and multivariate analysis of Overall Survival (OS) for EV-mediated *ELNAT1* expression in BCa patients (*n* = 242)

Variables	Univariate analysis			Multivariate analysis		
	**HR**	**95%CI**	***P*-value[i]**	**HR**	**95%CI**	***P*-value[i]**
Age (<65 vs. ≥65)	0.831	0.569-1.214	0.339			
Gender (Male vs. Female)	1.121	0.726-1.730	0.605			
T stage (T2-4 vs. T1)	1.273	0.847-1.913	0.245			
T grade (High vs. Low)	0.856	0.565-1.297	0.463			
Lymphatic metastasis (positive vs. negative)	1.580	1.029-2.428	**0.037***	1.224	0.783-1.916	0.375
EV-mediated *ELNAT1* expression (High vs. Low)	2.167	1.475-3.183	**0.001****	2.065	1.383-3.083	**0.001****

Abbreviations: HR = hazard ratio; 95%CI =95% confidence interval; T stage = tumor stage; T grade = tumor grade. [i] Cox regression analysis, * P <0.05, ** P <0.01.

Supplemental Table 9. Univariate and multivariate analysis of Disease-Free Survival (DFS) for EV-mediated *ELNAT1* expression in BCa patients (*n* = 242)

Variables	Univariate analysis			Multivariate analysis		
	**HR**	**95%CI**	***P*-value[i]**	**HR**	**95%CI**	***P*-value[i]**
Age (<65 vs. ≥65)	0.849	0.600-1.201	0.356			
Gender (Male vs. Female)	1.072	0.723-1.590	0.728			
T stage (T2-4 vs. T1)	1.265	0.872-1.835	0.215			
T grade (High vs. Low)	0.921	0.628-1.351	0.673			
Lymphatic metastasis (positive vs. negative)	1.843	1.270-2.675	**0.001****	1.412	0.941-2.118	0.095
EV-mediated *ELNAT1* expression (High vs. Low)	1.879	1.329-2.656	**0.001****	1.728	1.203-2.484	**0.003****

Abbreviations: HR = hazard ratio; 95%CI = 95% confidence interval; T stage = tumor stage; T grade = tumor grade. [i]Cox regression analysis, $^{*}P$ <0.05, $^{**}P$ <0.01.

Supplemental Table 10. Diagnostic performance of EV-mediated *ELNAT1* expression, urine cytology and FISH analysis of urinary samples in BCa patients and controls

Methods	BCa (n=242) vs. controls (n=166)			LN positive (n=50) vs. LN negative (n=192)		
	**Sensitivity (95%CI)**	**Specificity (95%CI)**	**Accuracy (95%CI)**	**Sensitivity (95%CI)**	**Specificity (95%CI)**	**Accuracy (95%CI)**
EV-mediated *ELNAT1* [i]	0.85 (0.79-0.89)	0.70 (0.63-0.77)	0.79 (0.75-0.83)	0.72 (0.57-0.83)	0.86 (0.81-0.91)	0.83 (0.78-0.88)
Urine cytology	0.56 (0.49-0.62)	0.98 (0.94-1.00)	0.73 (0.68-0.77)	0.76 (0.62-0.86)	0.08 (0.05-0.13)	0.22 (0.17-0.28)
FISH	0.88 (0.84-0.92)	0.6 (0.52-0.67)	0.77 (0.73-0.81)	0.54 (0.39-0.68)	0.28 (0.22-0.35)	0.43 (0.37-0.49)

[i] Sensitivity and specificity by EV-mediated *ELNAT1* were obtained at the best cutoffs.

Supplemental Table 11. The assessment of LN status by urinary EV-mediated *ELNAT1* expression compared with CT in BCa patients (n = 242)

Pathological LN status	EV-mediated *ELNAT1*-reported LN (+)[i]			EV-mediated *ELNAT1*-reported LN (-)			Total
	CT-reported LN (+)	CT-reported LN (-)	Total	CT-reported LN (+)	CT-reported LN (-)	Total	
**LN (+)**	17	19	36	2	12	14	50
**LN (-)**	6	20	26	10	156	166	192
**Total**	23	39	62	12	168	180	242

Abbreviations: LN (+) = lymph node positive; LN (-) = lymph node negative. [i] The best cut off of urinary EV-mediated *ELNAT1* was used to distinguish LN-positive and LN-negative BCa.

Supplemental Table 12. Primer and probes used in the experiments.

Gene	Sequence (5'-3')	Application
ELNAT1	F: TGGCAAGAGACTTCCTGAGG<br>R: CAGAATGCCATGGTTTCCCC	qRT-PCR
PROX1	F: CAGCCCGAAAAGAACAGAAG<br>R: GGGTCTAGCTCGCACATCTC	qRT-PCR
U6	F: CTCGCTTCGGCAGCACA<br>R: AACGCTTCACGAATTTGCGT	qRT-PCR
18S rRNA	F: ACACGGACAGGATTGACAGA<br>R: GGACATCTAAGGGCATCACA	qRT-PCR
MALAT1	F: GCAGGGAGAATTGCGTCATT<br>R: TTCTTCGCCTTCCCGTACTT	qRT-PCR
SUMO1	F: ATCAAGCCTCAGTCCCCTTC<br>R: TGCTAGAACATCAGGGCCAA	qRT-PCR
SUMO2	F: GACGAAAAGCCCAAGGAAGG<br>R: CATTGATTGGTTGCCCGTCA	qRT-PCR
SUMO3	F: GCAAGCTGATGAAGGCCTAC<br>R: TCAACAGCAATGCGAGGATG	qRT-PCR
SAE1	F: GGTGGCTGTCTTTGTTCCAG<br>R: AGAAGGTGACAAGAGGCTCC	qRT-PCR
UBA2	F: GCTGCCCGAAACCATGTTAA<br>R: AGGAAAGGTTCTCTGGGTCG	qRT-PCR
UBC9	F: AATTCTTCGTCCTGAGGCCA<br>R: GCTTCCCATCTCTGTCCACT	qRT-PCR
CCNB1IP1	F: ACTGCCTGCTCTCACATCTT<br>R: TGTCCAACACGATCTCTGGT	qRT-PCR
SENP6	F: CACATGGACAGTCGCAAAGG<br>R: TGAAAGCGAGCACACAAACA	qRT-PCR
SENP2	F: ATTCCCATTCCAGCTGACCA<br>R: AACCAAAGGAAGGCAGGACT	qRT-PCR
TRIM22	F: GGAGGGGCAGAAGAGAGATG<br>R: ACATTCCTTGACCACCTCGT	qRT-PCR
SENP7	F: TCGTCTCACTGGTATCTCGC<br>R: TGGGAATCCTCTGCACTCAA	qRT-PCR
SOX18	F: AGAAGCGTCACTGTGGCAAA<br>R: TTATTGTGGCCTCTCCGTCC	qRT-PCR
VEGF-C	F: TGGGGAAGGAGTTTGGAGTC<br>R: GTTACTGGTTTGGGGCCTTG	qRT-PCR
VEGF-D	F: ACCTTCCATTCACACCAGCT<br>R: GACACCTGCCATTCCATGAC	qRT-PCR

NOTCH	F: GACCTCATCAACTCACACGC<br>R: GGTGTCTCCTCCCTGTTGTT	qRT-PCR
COUP-TFII	F: CTGTCCCCACTCACTGTCTT<br>R: CCAACACACACACACCCAAA	qRT-PCR
FOXC1	F: TCTTCCTTGCCTCTCACCTG<br>R: ACACTTTCTGGCGTTTGGTC	qRT-PCR
MAFB	F: CTTCGACCTGCTCAAGTTCG<br>R: CCTCGAGGTGTGTCTTCTGT	qRT-PCR
GAPDH	F: CATGAGAAGTATGACAACAGCCT<br>R: AGTCCTTCCACGATACCAAAGT	ChIRP
β-actin	F: GAAGCTAAGTCCTGCCCTCA<br>R: GGACGAGAGAAGACTGGAGG	ChIP
UBC9-P1	F: TGAGTGAGGGAGGGAATGAA<br>R: GACAGTCCGTTCGCCAAC	ChIRP
UBC9-P2	F: TGAGTGAGTGAGGGTGGC<br>R: CTCCCCTACTCCGTCATTCC	ChIRP
UBC9-P3	F: ATGAGTTTGTGAGAGTGGGA<br>R: CTCACTCTCACTCATTCCCG	ChIRP
UBC9-P4	F: CATAGTGCCCGCAGATGG<br>R: CCCCAGAATCCAGTCCCTG	ChIRP
UBC9-P5	F: GGGAGGGAATGAGTGAGGGA<br>R: AAACTCATTCCCTCCCTCACT	ChIRP
miR-196A	F: GGTAGGTAGTTTCATGTTGTTGGG<br>R: Universal primer	qRT-PCR
miR-320	F: GCAGAGAAAAGCTGGGTTGAG<br>R: Universal primer	qRT-PCR
miR-18A	F: GATAGCAGCACAGAAATATTGGC<br>R: Universal primer	qRT-PCR
SOX18-P1	F: CTTCTGAGAGGTCGCGGG<br>R: CCAGATATAGCGGCTCAGGG	ChIRP
SOX18-P2	F: GCAGCCTCCCCAGAACTT<br>R: AGAAAGGGCTGCTGATGAGG	ChIRP
SOX18-P3	F: GGTTTGGGAGTGAGGACAGA<br>R: CAACAGCCGGTAAGTCCTTC	ChIRP
SOX18-P4	F: AGGTCAGAGGGTCCAGGG<br>R: ACGGAGCCCACAAAGAGAAG	ChIRP
SOX18-P5	F: AGGTGGCGAGATTCTGACC<br>R: CCCCTTCCTACTCCATCAGG	ChIRP
ELNAT1-sgRNA1	AAAGGATCCTCTAGTAGCCA	CRISPR/Cas9

ELNAT1-sgRNA2	TCATCAAGACAGTAAATCCC	CRISPR/Cas9
ELNAT1 5'RACE	TGACGGTAGTTTCCCAAGTTT	Nested PCR<br>(Outer)
ELNAT1 5'RACE	GCTTCACCTTGGCAACCTTA	(Inner)
ELNAT1 3'RACE	AGTCTCAGGCCTTTAGTGATGA	(Outer)
ELNAT1 3'RACE	ATGCCGTCTTGTGTTTCCTC	(Inner)
si-hnRNPA1#1	sense: CAGCUGAGGAAGCUCUUCATT<br>antisense: UGAAGAGCUUCCUCAGCUGTT	si-RNA
si-hnRNPA1#2	Mixed:<br>rCrArArCrUrUrCrGrGrUrCrGrUrGrGrArGrGrAdTdT	si-RNA
si-UBC9#1	sense: GGGAUUGGUUUGGCAAGAATT<br>antisense: UUCUUGCCAAACCAAUCCCTT	si-RNA
si-UBC9#2	sense: CAAUGAACCUGAUGAACUGTT<br>antisense: CAGUUCAUCAGGUUCAUUGTT	si-RNA
si-*ELNAT1*#1	sense: CCAUGCGUUCUUUGGGCUUTT<br>antisense: UAACUGUCGAGUUCCAUGCTT	si-RNA
si-*ELNAT1*#2	sense: CCCAGUGUUGACUCACCAATT<br>antisense: UUGGUGAGUCAACACUGGGTT	si-RNA
si-SOX18#1	sense: GCAAGAAGCAGGCGCGCAATT<br>antisense: UUGCGCGCCUGCUUCUUGCTT	si-RNA
*ELNAT1*_odds1	CATCATCACTAAAGGCCTGA 3'-Biotin labeled	ChIRP
*ELNAT1*_odds2	CTTGCAGTCCCATCGATTTG 3'-Biotin labeled	ChIRP
ELNAT1 odds3	GTACTCCTCAACAGTCACAA 3'-Biotin labeled	ChIRP
*ELNAT1*_odds4	GAGGCACATCAGTTACGTTG 3'-Biotin labeled	ChIRP
*ELNAT1*_even1	GAACGCATGGCGATTACTTT 3'-Biotin labeled	ChIRP
*ELNAT1*_even2	CTGGGTATTTTCTGACCACT 3'-Biotin labeled	ChIRP
*ELNAT1*_even3	TACCTACTTGGGGAAACCAT 3'-Biotin labeled	ChIRP
ELNAT1 even4	CACACAGCACTTAACCAAGC 3'-Biotin labeled	ChIRP
ELNAT1	TGGCTTCTATGTCAACACGTA<br>5'-DIG labeled and 3'-DIG labeled	ISH
U6	CACGAATTTGCGTGTCATCCTT<br>5'-DIG labeled and 3'-DIG labeled	ISH
Scramble	GTGTAACACGTCTATACGCCCA<br>5'-DIG labeled and 3'-DIG labeled	ISH

Supplemental Methods

Electron microscopy analysis

Transmission electron microscope was used to identify the characteristics of EVs. The isolated EVs were placed on the grid for 60 minutes and fixed with 2.5% glutaraldehyde for 10 minutes. Subsequently, the EVs were incubated with uranyl acetate for another 5 minutes, followed by washing in PBS for three times. The grid was further imaged using a Hitachi transmission electron microscope.

Lentivirus infection and cell transfection

To construct the *ELNAT1* stable-overexpressing BCa cell lines, the lentivirus infection was conducted. Briefly, the *ELNAT1* gene was cloned into the pCDH-CMV-MCS-EF1-Puro lentivirus vector which was further transfected into the HEK-293T cells. Then, the virus was harvested and used to infect the UM-UC-3 cells, followed by the selection of puromycin (Sigma Aldrich) for 2 weeks.

As for cell transfection, 2×10^5 BCa cells per well were seeded into the 6-well plate and cultured for overnight. Then, the siRNAs or plasmids (Genepharma company, Shanghai, China) were transfected into the BCa cells with the assistance of lipofectamine 3000 (Invitrogen) according to the manufacturer's instructions. qRT-PCR was used to detect the transfecting efficiency.

RACE

To explore the full-length of *ELNAT1*, the 5' and 3' RACE assays were conducted using

the 5'/3' RACE Kit (Roche, USA, Cat#3353621001) according to the manufacturer's protocols. The *ELNAT1*-specific nested PCR primer sequences are listed in Supplemental Table 12. PCR amplification was done to acquire the gene specific RACE product, which was further purified in 1% agarose gel and subjected to bidirectional sequencing with specific primers.

RNA extraction and qRT-PCR assays

According to the manufacturer's instructions, the TRIzol reagent from Life Technologies was used to extract the total RNAs from cells, tissues, and EVs, which were further reverse transcribed to cDNA using the Prime ScriptTM RT Master Mix (Takara, Japan, Cat#RR390B). We subsequently conducted the real-time PCR using TBGreen II (Takara, Japan, Cat#RR820A) to analyze the expression of target RNA. GAPDH was used as an internal reference and the $2^{-\Delta\Delta CT}$ method was used to further analyze the data. All of the primer sequences are listed in Supplemental Table 12.

Western blotting analysis

The cells were lysed in RIPA lysis buffer (Pierce, IL, USA) supplemented with protease inhibitor (Roche, CA, USA) for 30 minutes on ice, followed by the centrifugation at 4°C, 12,000g for 30 minutes. The concentration of total proteins was counted using the BCA Protein Assay Kit (Thermo Fisher Scientific, MA, USA, Cat#23227) according to the manufacturer's instructions. Subsequently, the proteins, after heating denaturation, were separated by electrophoresis in 10% SDS polyacrylamide gels (SDS–PAGE), which was further transferred into the polyvinylidene fluoride membrane. The membrane was blocked

with 5% BSA for 1 hour at room temperature and incubated with primary antibodies at 4°C overnight. After washing three times with TBST, the membrane was incubated with a specific HRP-conjugated secondary antibody for 1 hour at room temperature, which was further detected through the ECL reagents according to the manufacturer's instructions.

FISH

The BCa cells were seeded into a confocal plate. After overnight incubation, the cells were fixed in 4% paraformaldehydeand permeabilized in 0.5% Triton X-100. Subsequently, the Alexa Fluor 555-labeled *ELNAT1* probes (Invitrogen, CA, USA) were added to hybridize with the BCa cells at 37°C overnight. DAPI was used to stain the cell nuclei and the Zeiss LAM 710 focal Microscope (Carl Zeiss AG, Germany) was used to capture images for further analysis.

Immunofluorescence

To perform the immunofluorescence assays, 3×10^4 BCa cells were seeded into the confocal plate and cultured for overnight. The cells were fixed in 4% formaldehyde for 15 minutes followed by permeabilization with 0.1% Triton X-100 for 20 minutes. Subsequently, goat serum was used to block the cells for 30 minutes and the primary antibody was added to incubate at 4°C for overnight. The cells were further incubated with the secondary antibody in darkness at room temperature for 1 hour, followed by the nuclei staining with DAPI. The LSM710 confocal microscope (Zeiss, Pleasanton, CA, USA) was used to capture the images.

Nuclear fractionation

To detect the subcellular location of *ELNAT1*, nuclear fractionation assays were performed according to the instructions of the PARIS Kit (Thermo Fisher, MA, USA, Cat#AM1921). 1×10^6 BCa cells were harvested and lysed in an ice-cold cell fractionation buffer for 10 minutes, followed by centrifugation at 500g for 5 minutes at 4°C to separate the cytoplasm and nucleus fraction. The acquired products were added to TRIzol reagent to extract the RNAs, respectively.

Tube formation assays

The FBS-free ECM and Matrigel (BD Biosciences, CA, USA) were mixed at a 2:1 ratio and coated into the 24-well chambers followed by incubation at 37°C overnight. Subsequently, 1×10^5 pre-treated HLECs per well were seeded into the Matrigel chambers and the equivalent PBS and isolated EVs were added into the chambers. Tube formation was observed using the inverted fluorescence microscope. The length of tubes was analyzed by ImageJ software.

Transwell assays

1×10^5 pre-treated HLECs resuspended in 200 μL FBS-free culture media were seeded into the upper chamber of Transwell insert (8 μm, Corning Costar Corp, USA). Subsequently, a total of 700 μL culture media containing 5% FBS was added to the lower chamber. After treating with the equivalent PBS or isolated EVs in the upper chamber, the HLECs were cultured for 15 hours. Then the cells were fixed with methanol and stained with 0.1% crystal violet for 15 minutes. The inverted fluorescence microscope was used to capture the image.

ChIP assays

ChIP assays were performed using the EZ-Magna ChIP A/G Kit (Millipore, MA, USA, Cat#17-408). BCa cells or HLECs treated with 10 μg/ml indicated EVs were harvested and fixed in 1% formaldehyde for 10 minutes, followed by the incubation of 10 × glycine for 5 minutes at room temperature. Subsequently, the cell lysis buffer supplemented with protease inhibitor was added to lyse the fixed cells, and the cell lysate was further sonicated into short fragments between 100 bp and 200 bp. The sheared chromatin was immunoprecipitated with the target antibody at 4°C overnight. The cross-linking DNA was eluted and analyzed by qRT-PCR.

IP and co-IP assays

IP and co-IP assays were performed using the co-Immunoprecipitation Kit (Thermo Fisher, MA, USA, Cat#26149). Briefly, BCa cells were lysed in the lysis buffer followed by the immunoprecipitation with an amine-reactive resin pre-coupled with target antibodies, and the normal rabbit IgG as a negative control, at 4℃ overnight. The proteins were eluted to be further analyzed by mass septectomy and western blot analysis.

CRISPR/Cas9-mediated gene deletion

Single guide RNAs (sgRNAs) targeted *ELNAT1* were designed*,* respectively, and inserted into lentiCRISPR v2 plasmid. The constructed plasmids were stably transfected into the cells to knockout the expression of *ELNAT1*. The individually cloned cells were further analyzed through qRT-PCR to determine the knockout efficiency.

RIP assays

The EZ-Magna RIP Kit (Millipore, MA, USA, Cat#17-700) was used for RIP assays. 2×10^7 BCa cells were harvested and lysed in the lysis buffer. The anti-hnRNPA1 antibody or rabbit IgG as the negative control pretreated magnetic beads were incubated with the lysate to acquire the immunoprecipitate. qRT-PCR analysis was performed to determine the precipitated RNAs, and U1 was used as a non-specific control.

Serial deletion analysis

Serial deletion analysis was conducted to detect the fragment of *ELNAT1* contributed to its direct interaction with hnRNPA1. The various truncated *ELNAT1* sequences were cloned into the pGSI vector and further used in the RNA pulldown assays as previously described.

Dual-luciferase reporter assays

The dual-luciferase reporter assays were performed to detect the epigenetic regulation of *ELNAT1* on the promoter of *UBC9* and *SOX18*. Briefly, we constructed the pGL3 reporter plasmids by inserting different fragments of *UBC9* and *SOX18* promoter into the upstream of the promoter region of the luciferase gene. Subsequently, the constructed pGL3 plasmids were transfected into HLECs pre-seeded into a 6-well plate and incubated for 48 hours. The cells were harvested and completely lysed in the lysis buffer, followed by the detection of firefly and renilla fluorescence through the Dual-Luciferase Reporter Assay System (Promega, USA, Cat#E1910).

FRET and CD spectroscopy

For FRET assays, we labeled the TFO with 5-carboxytetramethylrhodamine (TAMRA) and the TTS with 5-carboxyfluorescein (FAM), both of which were added into the binding buffer with a ratio of 1:5. After incubation at 55°C for 10 minutes and 37°C for 10 hours, the Molecular Device M5 Plate Reader was used to detect the 480 and 690 nm fluorescence wavelengths in different groups.

For CD spectroscopy, the equilibration of the mixture of TFO (2.2 μM) and TTS oligos (2.2 μM) with a ratio of 1:1 was conducted in the binding buffer at 30°C for 1 hour. Then the Chirascan spectrometer (Applied Photophysics) was used to measure fluorescence wavelengths. The Control ssRNA/TTS was used as the negative control while the *FENDRR* TFO/*PITX2* TTS as the positive control. The oligos for CD spectroscopy and FRET analysis are summarized in Supplemental Table 5.
